# Dynamics of quantum droplets in an external harmonic confinement

**DOI:** 10.1038/s41598-022-10468-6

**Published:** 2022-04-28

**Authors:** Maitri R. Pathak, Ajay Nath

**Affiliations:** Indian Institute of Information Technology Vadodara, Gandhinagar, Gujarat 382 028 India

**Keywords:** Optics and photonics, Physics

## Abstract

Recent theoretical and experimental results show that one-dimensional (1D) weakly interacting atomic Bose-Bose mixtures with repulsive interspecies mean field (MF) interaction are stabilized by attractive quadratic beyond-mean-field (BMF) effects into self-bound quantum droplet (QD) in free space. Here, we construct an exact analytical model to investigate the structure and dynamics of QDs in presence of external harmonic confinement by solving the 1D extended Gross–Pitäevskii equation (eGPE) with temporal variation of MF and BMF interactions. The model provides the analytical form of wavefunction, phase, MF and BMF nonlinearities. The generation of QDs and interesting droplet to soliton transition in presence of regular/expulsive parabolic traps by taking the comparable MF and BMF interactions are illustrated. We derive the phase diagram of the droplet-soliton phase transition between amplitude of MF, BMF interactions and harmonic oscillator frequency. The strength and form of oscillator frequency are identified as key parameter for tuning the compression, fragmentation and transport of droplets. Finally, the stability of the obtained solutions are confirmed from Vakhitov–Kolokolov (VK) criterion and are found stable.

## Introduction

The formation of droplets is one of the characteristic feature of classical liquids which results due to counter balancing of attractive van der Waal’s interactions with repulsive interactions generated from high density of its constituents^1^. Recently, in one of the pioneering experiment in the field of Bose–Einstein condensate (BEC) and superfluids, a new type of dilute quantum droplets (QDs) are observed in Bose-Bose mixtures with isotropic contact interactions^2,3^ and anisotropic dipolar interactions^4,5^. Different from the classical liquids, these QDs are generated at very low temperatures ($10^{9}$ order less than classical liquids) in BEC phase which makes them ultradilute quantum liquid with its density $10^{8}$ orders of magnitude lower than liquid helium’s^6^. Although, the formation of liquid state in weakly interacting BEC is not possible according to mean-field theory, however, Petrov pointed out that the QDs phase can be stabilized when the dominant contribution to the energy due to the contact mean field (MF) interaction is close to vanishing and becomes comparable to the beyond mean field (BMF) effects Lee-Huang-Yang (LHY) corrections arising from quantum fluctuations^7^. Based on this stabilization proposal, the dilute QDs are experimentally realized in binary Bose-Bose mixtures^8^, dipolar gases^9^ and similar stabilization mechanisms are proposed for its existence in Bose-Fermi mixtures^10,11^ and dipolar mixtures^12,13^. These developments led to a plethora of works in this field^14–21^. It is relevant to mention that the formation of vortices in droplets^22,23^, multiple droplets^24^, existence of striped states^25^, droplet clusters^26^ and supersolid properties in phase coherent droplets^27^, are also predicted in BECs. The precise experimental control and tunability over the QDs parameters: population density of mixture constituents, inter- and intra- component interactions, confinement, temperature and spatial dimension, opens new direction in modelling the other complex many-body systems like liquid helium^6,21^. Further, one possible application of the QDs is considered in the precise designing of matter-wave interferometer^28^.

In this work, we aim to construct an exact analytical model for investigating the generation of QDs in cigar-shaped two component BEC mixture and study its spatio-temporal dynamics in the presence of external harmonic confinement. In order to construct the analytical model, we consider binary Bose-Bose mixture in one dimensional geometry. The choice of binary BECs offers the presence of competing intra- and inter-component interactions which is required for the generation and stabilization of QDs^7^ and these interactions are experimentally tunable using Feshbach resonance technique^29^. Additionally, the quasi 1D condensates are quite useful for studying fundamental physics problems and a variety of nonlinear excitations like solitons (bright, dark, grey), compactons, rogue waves, Faraday waves, etc. are theoretically and experimentally explored in it^30^. Further, the choice of 1D geometry offers significant stability advantage of nonlinear excitations in comparison to three-dimension geometry and a clean controllable many-body test bed for precise measurement of quantum many-body effects. Although, in recent times, the dynamics of QDs is investigated in 1D geometry in absence of any external confinement i.e. free space. Astrakharchik *et. al.* identified the two physically different regimes (Gaussian shape and broad flat top plateau) of QDs depending its size in free space using eGPE^31^ and Tylutki *et. al.* distinguished droplets from quasi-1D bright solitons by investigating the excitation spectrum of the QDs in free space^32^. However, the study of QDs in presence of external confinements got less attention in the current literature. The motivation for studying two-component BEC in presence of external harmonic trap is threefold: (i) study novel phase transition: by tuning interaction and particle number in binary bose-bose BEC mixture in the 1D configuration, Cheiney *et. al.* realized an interesting phenomena of droplet to bright soliton transition in the harmonic trap^3^ ; (ii) mimic actual experimental scenario: in the current QDs experiments, it’s difficult to completely realize the free space situation experimentally, as there is always a presence of weak external harmonic trapping potential due to residual trapping frequency along the axial direction^8^; and (iii) stability of droplets: presence of external harmonic trap not only might improve its stability but can find application as a bath for sympathetic cooling of other systems^7^. Therefore, theoretically it would be important to investigate the behavior of QDs in presence of external harmonic confinement. We calculate the non-trivial exact form of wavefunction, phase, MF and BMF nonlinearities. Although, a family of solutions are obtained for eGPE from the constructed model, we mainly emphasize on the localized non-linear solitary excitations. Further, we find that one of the consistency conditions governing the condensate dynamics and harmonic oscillator frequency interestingly maps with the linear Schrödinger equation and Riccati equation. This provides flat top droplet profile corresponding to each solvable quantum mechanical configuration and leads to a significant control over the system dynamics. Utilizing the nature of MF, BMF interactions and strength of oscillator frequency, we demonstrate the droplet to soliton transition in presence of regular/expulsive parabolic trap. We also identify that the oscillator frequency and the sign of MF, BMF interaction amplitude’s act as the key parameter for controlling the droplet-soliton transition in the system. Further, we investigate the fragmentation and merging of QDs by choosing the elliptic solution of eGPE. The temporal evolution of QDs is studied by estimating the number of droplets w.r.t. the oscillator frequency of trap. We also illustrate the transport of QDs corresponding to the variation of oscillator frequency in the droplet domain.

In the following section, we provide an exact analytical model for investigating the dynamics of QDs in a weakly interacting 1D mass-balanced binary Bose-Bose mixture in the presence of external harmonic trap using 1D eGPE. Here, we have considered the system with temporally varying competing repulsive cubic MF and attractive quadratic BMF interactions. The model and analytical framework for the calculated system variables is explained by finding the wavefunction, oscillator frequency, MF and BMF nonlinearities. It is shown that with suitable choice of the harmonic oscillator frequency, one can realize following experimentally realizable forms of trap configurations: (a) free space; (No confinement); (b) $V(x,t)=\frac{1}{2}M^2 x^2$; (regular harmonic); and (c) $V(x,t)=-\frac{1}{2}M^2 x^2$; (expulsive harmonic). Moreover, the the consistency conditions governing the oscillator frequency interestingly maps with the linear Schrödinger equation which leads to a significant temporal control over the droplet dynamics of the system. We then investigate the dynamics of condensate under the influence of regular and expulsive parabolic traps. In comparison to free space, the axial compression of the atomic number density is observed in above-mentioned confinement with the variation of oscillator frequency. We illustrate the droplet to soliton transition in the considered system and remarkably identify the strength of oscillator frequency and sign of MF, BMF interaction’s amplitude as the key parameters for realizing this transition. For a better insight of the phenomenon of droplet-soliton transition, a phase diagram between the MF, BMF interaction amplitude’s and the oscillator frequency is also plotted. Moreover, by choosing the elliptic solution of eGPE and oscillator frequency of trap, we achieve fragmentation, merging and transport of QDs across the potential. The stability of obtained solution is confirmed using Vakhitov–Kolokolov (VK) criterion through an estimation of slope of norm w.r.t. chemical potential.

## Results

### The model and analytical framework

We begin by considering the binary BEC with equal masses and equal number of atoms in the components under the influence of BMF (LHY corrections for quantum fluctuations) in presence of temporally varying external harmonic confinement. The same magnitude of equilibrium densities of binary mixture components makes the result analysis clearer and easier. In 1D geometry, the system is described by the following equation^14,33^:

1 $$\begin{aligned} i \hbar \frac{\partial \psi _1}{\partial t} + \frac{\hbar ^2}{2m}\frac{\partial ^2\psi _1}{\partial x^2} - (\Lambda _s(t) |\psi _1|^2 + \Lambda _c(t) |\psi _2|^2)\psi _1 + \Gamma (t) (|\psi _1|^2+|\psi _2|^2)^{1/2}\psi _1-V(x,t) \psi _1= & {} 0, \end{aligned}$$

2 $$\begin{aligned} i \hbar \frac{\partial \psi _2}{\partial t} + \frac{\hbar ^2}{2m}\frac{\partial ^2\psi _2}{\partial x^2} - (\Lambda _c (t) |\psi _1|^2 + \Lambda _s (t) |\psi _2|^2)\psi _2 + \Gamma (t)(|\psi _1|^2+|\psi _2|^2)^{1/2}\psi _2 -V(x,t) \psi _2= & {} 0, \end{aligned}$$

where $\psi _1$ ($\psi _2$) are the wavefunctions of first and second component of binary mixture. Further, we assume that the coupling constants describing the repulsion between the atoms in each components are equal: $g_{\uparrow \uparrow } = g_{\downarrow \downarrow }\equiv g=2 \hbar ^{2} a_{s}(t)/(m a^{2}_{\perp })$ and $g_{c}= g_{\uparrow \downarrow }$. Here, $\Lambda _s (t)=(g_{c}+3g)/2$ represents the self interaction coefficients whereas $\Lambda _c (t)=(g_{c}-g)/2$ is the cross interaction coefficients along with $\Gamma (t)=\sqrt{m}g^{3/2}/(\pi \hbar )$^33^. We also consider binary mixture under influence of an external harmonic trapping potential *V*(*x*, *t*) with frequency $\omega _{T}$ , i.e., $m \omega ^{2}_{T} x^{2}/2$. $a_{\perp }$ is the transverse oscillator length of the trap and $a_{s}(t)$ represent the time dependent inter-and intra- components atomic scattering lengths which is tunable through Feshbach resonance technique^29^.

Considering the binary mixture as mutually symmetric spinor components with $\psi _{1}=\psi _{2}=\psi _{0} \psi$, then the dynamics of the considered system reduces to the time-dependent dimensionless single eGPE^14^:

3 $$\begin{aligned} \left[ i\frac{ \partial }{\partial t} + \frac{1}{2} \frac{ \partial ^2}{\partial x^2} + g_1(t) |\psi | - g_2(t)|\psi |^2- \frac{1}{2}M(t)x^2=0 \right] \psi . \end{aligned}$$

Here, $\psi (x,t)$ can be treated as the condensate wave function of the droplet with mass *m* and $|\psi (x,t)|^{2}$ represents the normalized density of each component. The parameters $g_{1}(t)=\Gamma (t)$ and $g_{2}(t)=\Lambda _{s}(t) + \Lambda _{c}(t)$ are the binary mixture components coupling constants representing BMF and MF interactions. In experiments it is possible to tune $g_{1}(t)$ and $g_{2}(t)$, both to positive or negative values by changing the sign of atomic scattering length. Here, $g_{1}(t)$ and $g_{2}(t)$ are non-zero functions. Further, *M*(*t*) is the time dependent harmonic oscillator frequency; a constant *M*(*t*) implies an oscillator potential which can be confining or expulsive for $M>0$ or $M<0$, respectively. Here, the value of scaling parameters $x_0$, $t_0$, $\psi _0$ are $\frac{\hbar g_1(t)}{\Gamma (t)} \sqrt{\frac{\Lambda _s + \Lambda _c}{2m g_2(t)}}$, $\frac{\hbar (\Lambda _s + \Lambda _c) g_1^2(t)}{2 g_2 (t) \Gamma ^{2}(t)}$, $\frac{\sqrt{2} g_2(t) \Gamma (t)}{(\Lambda _s + \Lambda _c) g_1(t)}$, respectively^31^. Equation (3) is the extended GPE added with external harmonic confinement and for $M(t)=0$, it has been extensively used in the past theoretical studies to describe the structure and dynamics of quantum droplets^7,21^.

In order to obtain analytical solutions of (3) in presence of of the time-dependent harmonic trapping potential and discuss QDs spatio-temporal dynamics in it, we assume the following ansatz solution:

4 $$\begin{aligned} \psi (x,t)=\sqrt{A(t)}F[\xi (x,t)] e^{i\phi (x,t)}, \end{aligned}$$

where *A*(*t*) and $\xi (x,t)=\gamma (t) x + \delta$ are the time-modulated amplitude and travelling coordinate of the condensate, respectively. Here, $\gamma (t)$ is the positive definite functions of time with $\delta >0$. In principle, the $\gamma (t)$ and $-\delta /\gamma (t)$ represents the inverse of the width and the position of the center of mass of the excitation^34^, respectively. Further, we assume the quadratic form of the phase as:

5 $$\begin{aligned} \phi (x,t)=-\frac{\gamma '(t)}{2\gamma (t)} z^{2} -\int \frac{E \gamma ^2(t)}{2} \partial t. \end{aligned}$$

Now, by substitution of the ansatz Eq. (4) and phase form (5) in Eq. (3), we obtain the following consistency conditions:

6 $$\begin{aligned} -\frac{\partial ^2 F}{\partial \xi ^2}-G_1\mid F(\xi )\mid F(\xi ) +G_2\mid F(\xi )\mid ^{2} F(\xi )=E F(\xi ), \end{aligned}$$

where *E* is eigenvalue of Eq. (6), $G_1$, $G_2$ denote nonlinearity constant which can have positive or negative depending on scattering length along with:

7 $$\begin{aligned}&A(t)=\gamma (t), 2 g_{2}(t)-G_{2} \gamma (t) =0 , \end{aligned}$$

8 $$\begin{aligned}&2 g_{1}(t)-G_{1} [\gamma (t)]^{\frac{3}{2}} =0, \end{aligned}$$

9 $$\begin{aligned}&M(t)= \frac{\gamma ''(t)\gamma (t)-2\gamma '^{2}(t)}{2\gamma ^{2}(t)}, \end{aligned}$$

where *M*(*t*) is the oscillator frequency of the harmonic confinement. It is important to note here that if $\gamma (t)$ is constant then $M(t)=0$ i.e. no external harmonic trap. The Eq. (9) can be transformed into the well-known linear form of Schrödinger equation by taking $\gamma (t)=\frac{1}{a(t)}$:

10 $$\begin{aligned} a''(t)+2a(t)M(t)=0. \end{aligned}$$

**Figure 1:**
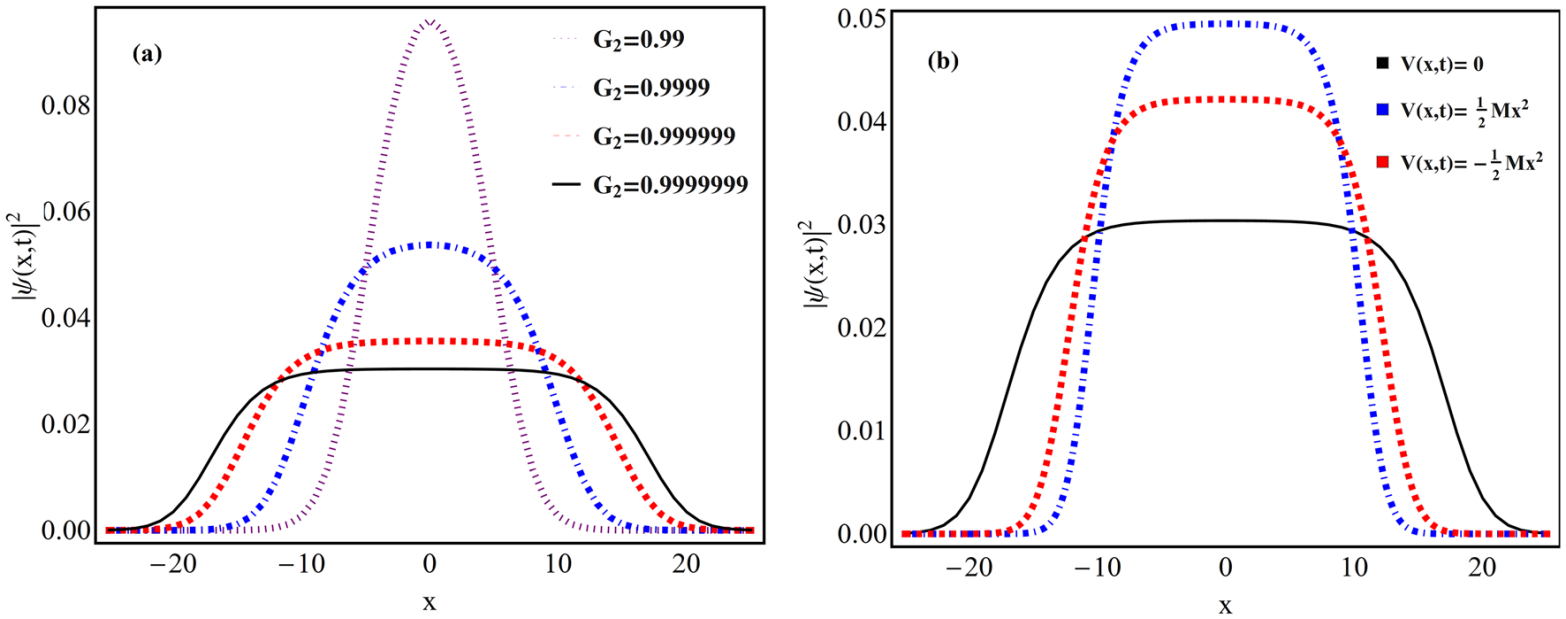
Quantum droplets in free space and external harmonic trap. Variation of density profiles (a) in free space w.r.t. changing magnitude of mean field interaction strength ($G_{2}$) for the physical parameters: $\gamma (t)=1$, $E=-2/9$, $G_{1} = -1$, along with $G_{2} = 0.99$ (dotted purple), 0.9999 (dotdashed blue), 0.999999 (dashed red) and 0.9999999 (thick black line); (b) in presence of regular harmonic trap (dotdashed blue line) and expulsive harmonic trap (dashed line red) for the physical parameter values: $\gamma _{0}=1.5$, $M=0.4$, $E=-2/9$, $G_{1} = -1$, $G_{2}=0.9999$ and $t=1$. Here, the free space quantum droplet profile is depicted with black line for $\gamma (t)=1$ and $G_{2}=0.9999$.

Equation (10) reveals a non-trivial correlation between the oscillator frequency and the amplitude of condensate wavefunction. This is one of the main results of this paper. By taking advantage of this connection, one can in principle identify QDs corresponding to each solvable quantum-mechanical system. Further, the Eq. (10) can be transformed into the Riccati equation,

11 $$\begin{aligned} \nu '(t)-\nu ^{2}(t)=2M(t), \end{aligned}$$

with $a(t)=e^{-\int _{0}^{t}\nu (t')dt'}$. The merit of these correlations is that it provides freedom to control the dynamics of the QDs in a number of analytically tractable ways as the Schrödinger equation and the Riccati equation can be exactly solved for a variety of *M*(*t*). The solution of Eq. (6) can be given as^7,31^:

12 $$\begin{aligned} F[\xi ]=\frac{3 (E/G_{1}) }{1+\sqrt{1-\frac{E}{\mu _{0}} \frac{ G_{2}}{ G_{1}^{2}} } \cosh (\sqrt{\text {-E}}\xi )} \end{aligned}$$

where, $$\mu _{0}=-2/9$$ with $$ E<0$$, $$G_1<0$$ and $$G_2>0$$. Thus, the complete solution of Eq. (3) can be written as:

13 $$\begin{aligned} \psi (x,t)=\frac{3 (E/G_{1}) \sqrt{\gamma (t)} }{1+\sqrt{1-\frac{E}{\mu _{0}} \frac{ G_{2}}{ G_{1}^{2}} } \cosh (\sqrt{\text {-E}} (\gamma (t) x + \delta ))} e^{i\left[ -\frac{\gamma '(t)}{2\gamma (t)} x^{2} -\int \frac{E \gamma ^2(t)}{2} \partial t\right] }. \end{aligned}$$

Equation (13) represents the localized solution of Eq. (3). Below, we study the dynamics of QD’s by considering the exact solution (13) of eGPE in presence of temporally varying repulsive cubic MF and attractive quadratic BMF interactions in harmonic confinement. Based on Eq. (9), we investigate the droplet dynamics for the following experimentally relevant forms of harmonic traps with suitable choice of oscillator frequencies *M*(*t*) and $\gamma (t)$:

- Free space: $\gamma (t)=$constant, $M(t)=0$; No confinement
- Regular harmonic: $$\gamma (t)= \gamma _{0} sec(M t)$$, $$M(t)=M^2$$; $$V(x)=\frac{1}{2} M^2 x^2$$
- Expulsive harmonic: $$\gamma (t)= \gamma _{0} sech(M t)$$, $$M(t)=-M^2$$; $$V(x)=-\frac{1}{2} M^2 x^2$$

where, $\gamma _{0}$, and *M* are real positive constants. In principle, depending on the need of the physical situation, one can choose the suitable $\gamma (t)$ form for controlling the depth and width of the resultant traps. Here, in the following sections, we demonstrate the generation of QD’s in above mentioned trap configurations i.e. in absence/presence of regular/expulsive parabolic traps.

### Quantum droplets in free space with $M(t)=0$

In this section, we investigate the generation of QD in absence of external confinement i.e. free space. In the 1D geometry, the dynamics of QDs is extensively investigated in free space scenario^7,21,31^. Here, from the constructed analytical model, we write down the wavefunction solution of Eq. (3) for $V(x,t)=0$. For this, we consider $\gamma (t)=$constant $=\gamma$, which ensures that *M*(*t*) becomes zero from Eq. (9). Thus, the complete wavefunction solution form of Eq. (13) for $\delta =0$ becomes:

14 $$\begin{aligned} \psi (x,t)=\frac{3 (E/G_{1}) \sqrt{\gamma } }{1+\sqrt{1-\frac{E}{\mu _{0}} \frac{ G_{2}}{ G_{1}^{2}} } \cosh (\sqrt{\text {-E}} \times \gamma x )} e^{i\left[ -\int \frac{E \gamma ^2}{2} \partial t\right] }, \end{aligned}$$

where $$\gamma > 0$$, with $$ E<0$$, $$G_1<0$$ and $$G_2>0$$. The obtained form (14) of free space wavefunction solution for eGPE (4) is identical to the results reported in the literature with $$G_{1}=-1$$^7,21,31^.

Further, for this form of $\gamma (t)$, the BMF and MF interactions becomes: $g_{1}(t)=(1/2)G_{1}\gamma ^{\frac{3}{2}}$, and $g_{2}(t)=(1/2)G_{2}\gamma$, respectively. Depending upon the sign of $G_{2}$, Eq. (14) leads to investigation of QDs dynamics in three characteristic regimes: (a) $G_{2}>0$: flat top density profile i.e. droplet domain, (b) $G_{2}<0$: integrable GPE with bright soliton solution with quantum fluctution term approaching to zero, and (c) $G_{2}$ is very small: GPE with only quadratic nonlinearity giving rise to the droplet wave function of the Korteweg-de Vries-type^32^.

In Fig. 1a, we plot the variation of condensate density profiles for the wavefunction (14) w.r.t. the changing magnitude of mean field interaction i.e. $G_{2}$ with $G_{2}>0$. Here, the physical parameter values: $\gamma (t)=1$, $E=-2/9$, $G_{1} = -1$, along with $G_{2} = 0.99$ (dotted purple), 0.9999 (dotdashed blue), 0.999999 (dashed red) and 0.9999999 (thick black line). It is apparent from the figure that as MF interaction strength $G_{2} \rightarrow 1$ and becomes comparable to BMF interaction strength $G_{1}=-1$, the signature feature of QD’s i.e. flat top density profile is realized. The variation of condensate density with the MF interaction parameter $G_{2}$ is in conformity with the physical situations reported in the literature^32^.

**Figure 2:**
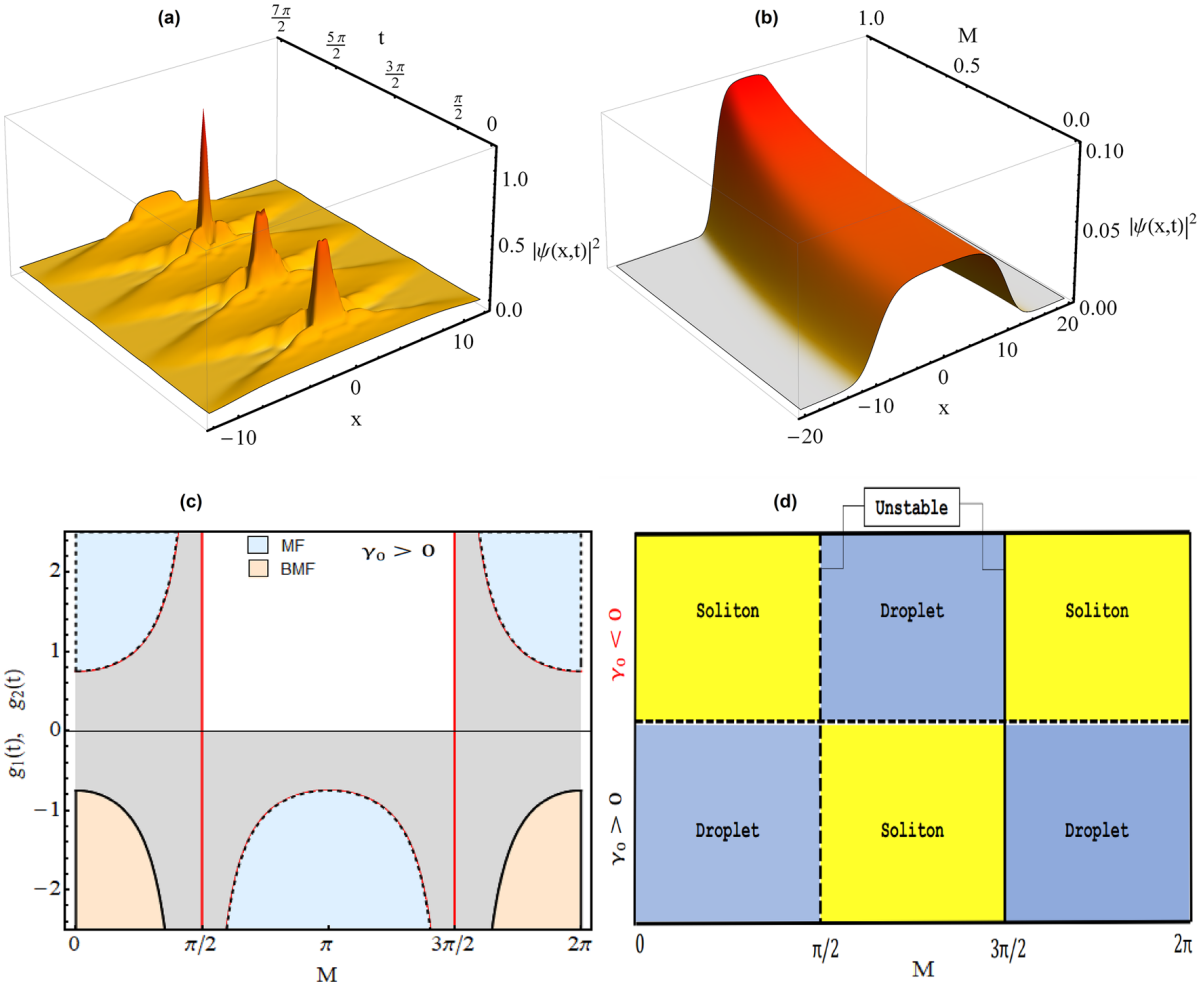
Quantum droplet to soliton transition in regular harmonic trap. The condensate densities are displayed with varying (a) time (*t*) with $M=1$; and (b) *M* with $t=1$. (c) Variation of corresponding MF ($g_{2}(t)$, dashed line) and BMF interactions ($g_{1}(t)$, thick line) w.r.t. *M* at $t=1$. (d) Phase diagram between $\gamma _{0}$ and *M* representing droplet to soliton transition. Here, other physical parameters are: $\gamma _{0}=1.5$, $E=-2/9$, $G_{1} = -1$, and $G_{2}=0.999999$. All parameters are in dimensionless unit. Figures are plotted using MATLAB R2020b (Master License 31349846) and Mathematica version 1.5.1.2021061827, Wolfram Research, Inc.

### Quantum droplets in regular harmonic trap with $M(t)=M^2$

In comparison to previous section, here we investigate the impact of regular harmonic trap on the dynamics of QDs. Although, the droplets are extensively explored in free space domain, however, its study had got less attention in presence of external harmonic traps. Here, from the constructed model, we calculate the form of wavefunction in presence of regular parabolic trap. In order to realize the chosen trap form, we take $\gamma (t)= \gamma _{0} sec(M t)$ which makes the form of oscillator frequencey: $M(t)= M^{2}$ from (9). Now, using the Eq. (13) with $\delta =0$, the resultant form of complete wavefunction for regular harmonic trap can be written as:

15 $$\begin{aligned} \psi (x,t)=\frac{3 (E/G_{1}) \sqrt{\gamma _{0} sec(M t)}}{1+\sqrt{1-\frac{\mu }{\mu _{0}} \frac{ G_{2}}{ G_{1}} } \cosh (\sqrt{\text {-E}} \times \gamma _{0} sec(M t) x )} e^{i\left[ -M \times Tan[M t] z^{2} -\int \frac{E \gamma ^2_{0} sec^2(M t)}{2} \partial t\right] }. \end{aligned}$$

with $$\gamma _{0}>0$$, $$M>0$$, $$ E<0$$, $$G_1<0$$ and $$G_2>0$$.

In Fig. 1b, we compare the profile of condensate densities for the regular harmonic trap (dotdashed blue line) with the free space domain (black line) using wavefunction solutions (14) and (15), respectively. For plotting, the magnitude of dimensionless physical parameters are: $\gamma _{0}=1.5$, $M=0.4$, $E=-2/9$, $G_{1} = -1$, $G_{2}=0.9999$ and $t=1$. Specifically, for comparison, the free space QD density profile (black line) is plotted with $\gamma (t)=1$ and $G_{2}=0.9999$. Figure clearly illustrate that in comparison to free space, QDs gets compressed due to the presence of regular parabolic trap which is as per physical understanding.

In Fig. 2, we illustrate an interesting physical phenomena of droplet to soliton transition in regular harmonic trap by tuning the strength of MF and BMF interactions. Recently, Cheiney *et. al.* reported the bright soliton to QD transition in a binary mixture of two component BEC in presence of external harmonic confinement by tuning the interaction strength and particle number^3^. Motivated from this, in Fig. 2a, we investigate the spatio-temporal dynamics of QD in chosen trap configuration with $g_{1}(t)=(1/2)G_{1}[\gamma _{0} sec(M t)]^{\frac{3}{2}}$, and $g_{2}(t)=(1/2)G_{2} \gamma _{0} sec(M t)$ i.e. $\gamma (t)=\gamma _{0} sec(M t)$ for $M=1$. It is evident from figure that initially, at $t=0$, the condensate density having QD profile which with increase in time demonstrate strong compression at $t=n \pi /2$, where *n* is a real number, and these condensate atoms coalesces forming large density peaks. Here, other physical parameters are: $\gamma _{0}=1.5$, $E=-2/9$, $G_{1} = -1$, and $G_{2}=0.999999$. This behavior of condensate density can be understood by plotting the $g_{1}(t)$ (thick line) and $g_{2}(t)$ (dashed line) profiles w.r.t. *M* at $t=1$ (Fig. 2c) for the same parameter values. It shows that the magnitude of $g_{2}(t)$ and $g_{1}(t)$ increases sharply near $n \pi /2$ points due to presence of *sec*(*Mt*) term which physically represents a strong repulsive MF and attractive BMF interactions. Further, the magnitude of $g_{1}(t)$ is proportional to $[\gamma _{0} sec(M t)]^{\frac{3}{2}}$ leading to compression of condensate atomic density. Analytically, the sign of $g_{1}(t)$ and $g_{2}(t)$ changes at $n \pi /2$ values i.e. in the $[0,\pi /2]$ and $[3\pi /2,2\pi ]$ domains, $g_{2}(t)$ is positive whereas in between $[\pi /2,3\pi /2]$, this becomes negative whereas $g_{1}(t)$ is negative in $[0,\pi /2]$ and $[3\pi /2,2\pi ]$. The change of sign $g_{2}(t)$ results in making MF interactions repulsive to attractive with variation of *M*. As the balance of MF and BMF interaction is required for realizing QD and as discussed earlier, for $g_2(t)>0$, the density profile of Eq. (3) will be droplet whereas if $g_{2}(t) <0$, then it becomes soliton^7,32,35^. In the time interval $[\pi /2, 3\pi /2]$, the considered system will have attractive MF interaction with continuous loss of condensate atoms due to imaginary BMF term leading to formation of bright solitons^36^. On this basis, we reveal an analytical scenario for the droplet to soliton in regular harmonic trap with the change in sign of $\pm g_{2}(t)$ in Fig. 2c at $n\pi /2$ points. Based on this, in Fig. 2d, we construct a phase diagram in between $\gamma _{0}$ i.e. amplitude of MF, BMF interaction’s and *M*. For $\gamma _{0}>0$, $g_{2}(t)$ is positive in $[0,\pi /2]$ and $[3\pi /2,2\pi ]$ domains i.e. droplet density profile whereas it becomes negative in between $[0,\pi /2]$ with $\gamma _{0}<0$ resulting into soliton formation. This is one of the important result of this article revealing non-trivial correlation between the droplet to soliton phase transition with *M* and $\gamma _{0}$. Here, all the parameters are in dimensionless unit with magnitude: $\gamma _{0}=1.5$, $E=-2/9$, $G_{1} = -1$, and $G_{2}=0.999999$. Further, in Fig. 2b, we illustrate the impact of the strength of *M* on single QD profile using (15). It is apparent from the Fig. 2b that with *M* tending from 0 $\rightarrow$ 1 leads to increase in height and decrease in the width of QDs profile due to its compression. In principle, by tuning the oscillator frequency of external confinement, one can tune the width and height of QDs. Here, we depicted the profile with physical parameters: $\gamma _{0}=1.5$, $t=1$, $G_{2}=0.999999$, $E=-2/9$, $G_{1} = -1$.

*Fragmentation and merging of QDs by tuning oscillator frequency:* We demonstrate a novel mechanism for the fragmentation and merging of QDs with the tuning of oscillator frequency of regular harmonic trap. We show that by choosing the elliptic solution of Eq. (6) in place of localized solution, one can induce the fragmentation in QDs. In order to demonstrate this effect, we write the solution of Eq. (6) as:

16 $$\begin{aligned} F(\xi )= B \;\; Cn[\beta \; \xi ,\;q]+D, \end{aligned}$$

where $B=\sqrt{\frac{2}{(2q^{2}-1)}}D$ $>0$, $D=\frac{G_{1}}{3G_{2}}$ $<0$, and $\beta ^{2}=-(\frac{6G_{2}}{(2q^{2}-1)})$ $D^{2}$, and $q^{2}>1/2$^19^. Here, *q* is the modulus parameter for the Jacobi elliptic function *cn*. For investigating the dynamics of condensate in the regular harmonic confinement, we take the same parameter values of Fig. 2 i.e. $\gamma _{0}=1.5$, $E=-2/9$, with $\gamma (t)=\gamma _{0} \sec (M t)$. Thus, using this, the complete wavefunction solution of (13) becomes:

17 $$\begin{aligned} \psi (x,t)=3 (E/G_{1}) \sqrt{\gamma _{0} sec(M t)} \; \left[ B\; Cn(\beta \;\xi ,\;q)+D \right] e^{i\left[ -M \times Tan[M t] z^{2} -\int \frac{E \gamma ^2_{0} sec^2(M t)}{2} \partial t\right] } \end{aligned}$$

with $$\delta =0$$, $$M>0$$ with $$ E<0$$, $$G_1<0$$ and $$G_2>0$$.

**Figure 3:**
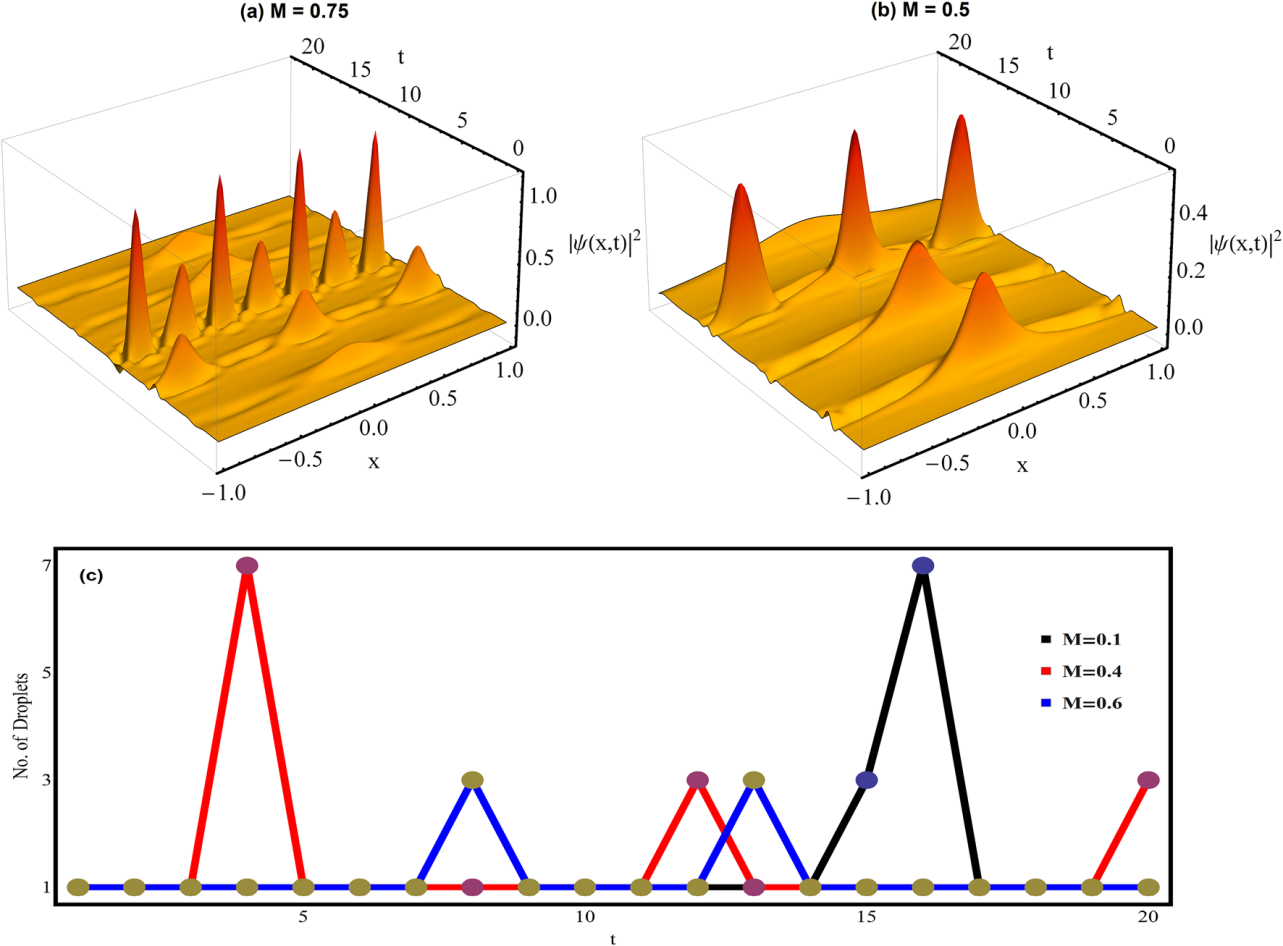
Fragmentation and merging of quantum droplets in regular harmonic trap. The condensate density profiles are plotted for (a) $M=0.75$ and (b) $M=0.5$ with other parameters values: $\gamma _{0}=1.5$, $E=-2/9$, $G_{1} = -1$, $G_{2}=0.999999$ and $q=0.9$. (c) The number of droplets w.r.t. time *t* is depicted for $M=0.1$ (black line), $M=0.4$ (red line), and $M=0.6$ (blue line). Here, all the parameters are made dimensionless. Figures are plotted using MATLAB R2020b (Master License 31349846) and Mathematica version 1.5.1.2021061827, Wolfram Research, Inc.

Figure 3a and b depicts the impact of magnitude of *M* on the fragmentation and merging of QDs for $M=0.75$ and $M=0.5$ with $q=0.9$. Here, we have taken comparable strength of MF and BMF interaction strengths with $G_{1} = -1$, and $G_{2}=0.999999$, respectively so that density profile remains in droplet regime (Fig. 2b). For exploring the condensate dynamics, we considered potential in the range $[-1, 1]$. It is evident from the figures that, initially at $t=0$, a single QD profile is observed and with increase in time, it gets fragmented and forms the QD train. Further, with increase in time, these fragmented components starts to coalesce and again merges to form a single QD. Importantly, with *M* changing from $0.5 \rightarrow 0.75$, the number of fragmented droplets increase. In Fig. 3c, we have separately depicted the number of droplets at different instant of time for $M=0.1$ (black line), $M=0.4$ (red line), and $M=0.6$ (blue line), respectively. It is quite clear that with increase in magnitude of *M* from $0.1 \rightarrow 0.6$, the time of fragmentation reduces but along with that number of droplets formed also decreases. In cigar shaped BEC, the bright solitary trains are experimentally explored in harmonic trap^38^ and through tuning of condensate atoms gain profile theoretically investigated by Atre *et. al.*^39^. Recently, Cidrim *et. al.* explored the generation of soliton trains in two component BEC^40^. The present temporal evolution in Fig. 3 allows us to obtain fragmentation and merging of QDs by tuning of oscillator frequency and persisting for future experimental realization.

**Figure 4:**
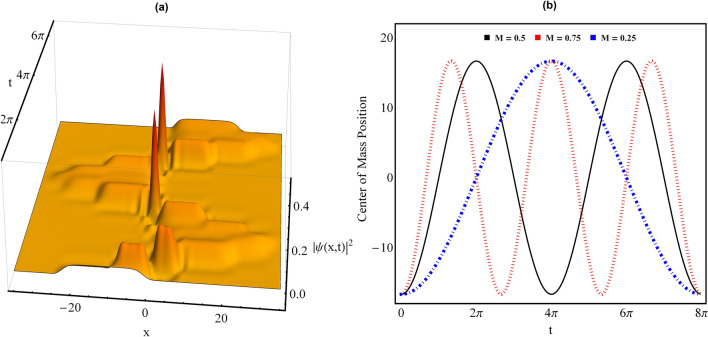
Transport of quantum droplets in regular harmonic trap. (a) Condensate density profile for $M=0.5$, and (b) the center of mass position of quantum droplet are depicted for the parameter values: $\gamma _{0}=1.5$, $\delta =25$, $E=-2/9$, $G_{1} = -1$, and $G_{2}=0.999999999$. In (b), black thick line ($M=0.5$), dotted red line ($M=0.75$), and blue dotdashed line ($M=0.25$). Here, all the parameters are in dimensionless units. Figures are plotted using MATLAB R2020b (Master License 31349846) and Mathematica version 1.5.1.2021061827, Wolfram Research, Inc.

*Transport of droplets across potential:* In Fig. 4a, we illustrate an analytical scenario for the oscillation and transport of QDs in regular harmonic confinement in the considered system. In order to realize the transport of QDs in parabolic trap, we have taken $\delta \ne 0$ in Eq. (13) along with $\gamma (t)= \gamma _{0} sec(M t)$ which ensures the form of trap as $(1/2) M^{2}x^{2}$. Thus, the resultant wavefunction becomes:

18 $$\begin{aligned} \psi (x,t)=\frac{3 (E/G_{1}) \sqrt{\gamma _{0} sec(M t)}}{1+\sqrt{1-\frac{E}{\mu _{0}} \frac{ G_{2}}{ G_{1}^{2}} } \cosh (\sqrt{\text {-E}} \times (\gamma _{0} sec(M t) x +\delta ) )} e^{i\left[ -M Tan[M t] z^{2} -\int \frac{E \gamma ^2_{0} sec^2(M t)}{2} \partial t\right] }, \end{aligned}$$

with $$\gamma _{0}>0$$, $$M>0$$, $$ E<0$$, $$G_1<0$$ and $$G_2>0$$.

In Fig. 4a, we plot the condensate density profile for wavefunction solution (18) for the parameter values: $\gamma _{0}=1.5$, $\delta =25$, $E=-2/9$, $G_{1} = -1$, $G_{2}=0.999999999$ and $M=0.5$. Figure clearly depicts the oscillation of QD across the chosen harmonic trap. In principle, the center of mass position of condensate density is connected with [$-\delta /\gamma ((t)$] and is oscillating w.r.t. time^37^. For the considered case, $-\delta /\gamma ((t)$ $=-(\delta /\gamma _{0}) \cos (M t)$ i.e. for $M=0.5$, the maximum magnitude of $-(\delta /\gamma _{0}) \cos (M t)$ appears at $t=2n \pi$. This is validated from Fig. 4a as the direction of oscillation of QDs changes at $t=2n \pi$ locations. For providing a better interpretation of the oscillation of QDs in this system, we plot $-(\delta /\gamma _{0}) \cos (M t)$ w.r.t. *M* in Fig. 4b for the same parameter values: $\gamma _{0}=1.5$, $\delta =25$, $E=-2/9$, $G_{1} = -1$, and $G_{2}=0.999999999$. Here, $M=0.5$ (black thick line), $M=0.75$ (dotted red line), and $M=0.25$ (blue dotdashed line). It is apparent from the Fig. 4b that as *M* changes from $0.5 \rightarrow 0.75$, then temporal periodicity of oscillation increases whereas with *M* becoming $0.5 \rightarrow 0.25$ leads to its decrease. Thus, we reveal a non-trivial analytical connection of oscillator frequency with the transport and oscillation of QDs and by using this, one can control the temporal periodicity of QDs oscillation.

### Quantum droplets in expulsive harmonic trap with $M(t)=-M^2$

In this section, we study the generation of QDs in presence of expulsive harmonic confinement and the droplet to soliton transition. In order to investigate the dynamics of QDs in presence of expulsive harmonic trap, we take $\gamma (t)= \gamma _{0} sech(M t)$ in Eq. (6) which results into the trap form $-(1/2) M^{2}x^{2}$. Substituting this form of $\gamma (t)$ in Eq. (13) gives the wavefunction for expulsive parabolic trap as:

19 $$\begin{aligned} \psi (x,t)=\frac{3 (E/G_{1}) \sqrt{\gamma _{0} sech(M t)}}{1+\sqrt{1-\frac{E}{\mu _{0}} \frac{ G_{2}}{ G_{1}} } \cosh (\sqrt{\text {-E}} \times \gamma _{0} sech(M t) x)} e^{i\left[ M \times Tanh[M t] z^{2} -\int \frac{E \times \gamma ^2_{0} sech^2(M t)}{2} \partial t\right] } . \end{aligned}$$

with $$\delta =0$$, $$\gamma _{0}>0$$, $$M>0$$, $$ E<0$$, $$G_1<0$$ and $$G_2>0$$.

**Figure 5:**
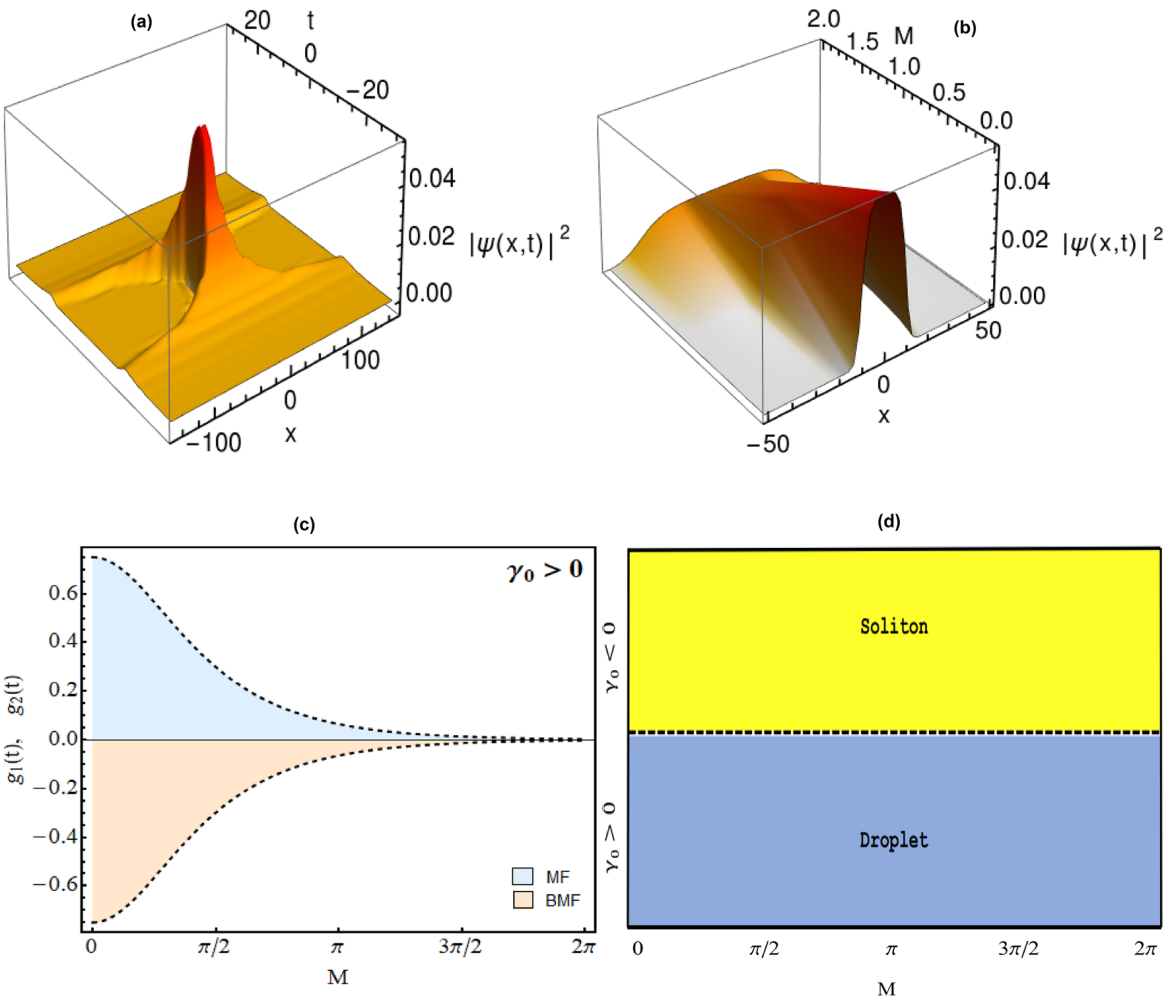
Quantum droplet dynamics in expulsive harmonic trap. The compression of condensate density w.r.t. (a) time (*t*) for $M=0.25$, and (b) oscillator frequency *M* at $t=1$ for the dimensionless parameter values: $\gamma _{0}=1.5$, $E=-2/9$, $G_{1} = -1$, $G_{2}=0.999999$. For the same parameter values, the variation of $g_{1}(t)$ (light orange filled region) and $g_{2}(t)$ (light blue filled region) w.r.t. *M* at $t=1$ are depicted in (c). (d) Phase diagram representing the droplet to soliton transition in between $\gamma _{0}$ and *M* in presence of expulsive trap. Figures are plotted using MATLAB R2020b (Master License 31349846) and Mathematica version 1.5.1.2021061827, Wolfram Research, Inc.

In Fig. 1b, we depict the profile of condensate density for expulsive harmonic trap (dashed line red) for the physical parameter values: $\gamma _{0}=1.5$, $M=0.4$, $E=-2/9$, $G_{1} = -1$, $G_{2}=0.9999$ and $t=1$. Here, for comparison, quantum droplet profile in presence of the free space is also depicted with black line for $\gamma (t)=1$ and $G_{2}=0.9999$. The presence of expulsive trap results in the increase of height and decrease in droplet width in comparison to free space droplet profile (black line) and this is as per physical understanding. Further, in Fig. 5, we investigate the spatio-temporal dynamics of condensate density in presence of expulsive harmonic trap. Figure 5a depicts the temporal evolution of droplet for $M=0.25$ and clearly reveal its compression at $t=0$. Here, we considered the parameter values: $\gamma _{0}=1.5$, $E=-2/9$, $G_{1} = -1$, $G_{2}=0.999999$. This compression of droplet in expulsive trap can be understood from the MF and BMF interactions profile represented in Fig. 5c. For $\gamma (t)=\gamma _{0} sech(Mt)$, the form of BMF and MF interaction becomes: $g_{1}(t)=(1/2)G_{1} [\gamma _{0} sech(M t)]^{\frac{3}{2}}$, and $g_{2}(t)=(1/2)G_{2} \gamma _{0} sech(M t)$, respectively. It is apparent from Fig. 5c that $g_{2}(t)$ and $g_{1}(t)$ have maximum magnitude at $M=0$ and $t=1$ i.e. strong repulsive and attractive interactions whereas their amplitude decreases with increase in *M* for $\gamma _{0}>0$. However, $g_{1}(t)$ is proportional to $[\gamma _{0} sech(M t)]^{\frac{3}{2}}$ leading to strong compression. Similar, temporal profile of $g_{1}(t)$, $g_{2}(t)$ can be obtained by keeping $M=1$ and varying time. Fig. 5c clearly justifies the strong compression in condensate density at $t=0$ in Fig. 5a. It is important to note here that the profile of Fig. 5c get reversed for $\gamma _{0} <0$ which means $g_{2}(t)$ becoming attractive with continuous loss of condensate atoms due to imaginary BMF term leading to formation of bright solitons^36^. As discussed above, in this domain, the condensate density becomes soliton. Based on this, we construct a phase diagram in between $\gamma _{0}$ and *M* in Fig. 5d which shows that droplet to soliton transition in expulsive trap can be observed by changing the sign of MF and BMF interaction’s amplitude. Different from regular harmonic confinement, the droplet to soliton transition occurs in expulsive trap by tuning the sign of MF and BMF interactions. It is due to the change in nature of MF, BMF interactions from $\sec (Mt)$ (regular harmonic) to *sech*(*Mt*) (expulsive harmonic). Figure 5b illustrates the variation of droplet profile w.r.t. changing magnitude of *M*. With increase in magnitude of *M* from 0 $\rightarrow$ 1 leads to decrease in height and increase in the width of QDs profile due to its expansion with changing strength of oscillator frequency. In principle, by tuning the oscillator frequency of external confinement, one can tune the width and height of QDs. Here, we depicted the profile with physical parameters: $\gamma _{0}=1.5$, $E=-2/9$, $G_{1} = -1$, $G_{2}=0.999999$ and $t=1$.

**Figure 6:**
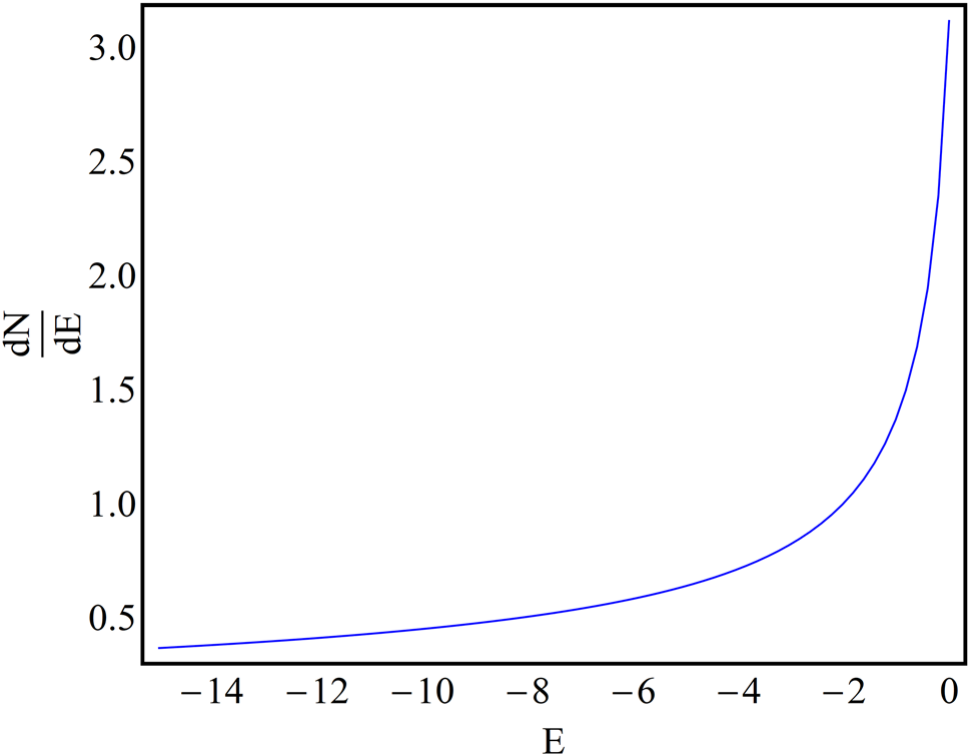
Stability analysis of obtained solution. The slope of norm *N* w.r.t. *E* is plotted as a function of chemical potential (*E*) diagrams as a function of the *E* Vs with $G_{1} =-1$.

### Stability analysis

Using various forms of $\gamma (t)$ in Eq. (13), we investigated the generation and spatio-temporal dynamics of droplets in absence/presence of regular/expulsive traps. In this section, we perform the stability analysis of (13) using the VK criterion^41^. The VK criterion is widely used for the stability analysis of nonlinear Schrödinger equation and it is already established in the literature that necessary condition for a solution to be stable is positive slope of *dN*/*dE*, where *N* is the normalization and *E* is the chemical potential of the system. If the *dN*/*dE* of obtained solution is negative then the solution is unstable and for the condition that ${dN}/{dE} = 0$, the solution remains marginally stable. Now, using Eq. (13) and $N=\int _{-\infty }^{+\infty }|\psi |^2 \partial x$, one can estimate the correlation between normalization *N* and *E* as:

20 $$\begin{aligned} N=\frac{4}{3} \left[ ln \left( \frac{1+\sqrt{\frac{E}{\mu _{0}}}}{\sqrt{1-\frac{E}{\mu _{0}}}}\right) -\sqrt{\frac{E}{\mu _{0}}}\right] , \end{aligned}$$

where $G_{1}=-1$ and $\frac{ G_{2}}{ G_{1}^{2}} \approx 1$. Equation (20) estimates the magnitude of *N* in presence of harmonic trap and is equal to the *N* reported for free space^31,32^. Thus, even in the presence of external trap, the system has a continuous symmetry property and *N* is conserved in time as per Noether’s theorem^42^. In Fig. 6, using Eq. (20), we plot *dN*/*dE* w.r.t. *E* where $G_{1}=-1$. It is evident from the figure, that the magnitude of *dN*/*dE* is positive w.r.t. its variation *E* which indicates the stable nature of the obtained solution.

## Summary

The main results reported in this paper are summarized as follows. We report a large family of exact analytical solution of 1D eGPE for investigating the structure and dynamics of QDs in harmonic confinement for temporally varying MF and BMF interactions. We have considered a weakly interacting 1D mass-balanced binary Bose-Bose mixture with competing repulsive cubic MF and attractive quadratic BMF interactions. The dynamics of QDs investigated w.r.t. the oscillator frequency and the strength of BMF interaction in different experimentally realizable forms of trap configurations: (a) $V(x,t)=\frac{1}{2}M^2 x^2$; (regular harmonic); and (b) $V(x,t)=-\frac{1}{2}M^2 x^2$; (expulsive harmonic). The constructed solutions for the free space is in agreement with existing results in the current literature^31^. Compression and flat top profile of the atomic number density is illustrated in above-mentioned confinement with the variation of *M* and $G_{2}$. Such flat top density profile was reported in the literature for QDs in free space domain. We have observed the droplet to soliton transition in regular/expulsive harmonic confinement depending on the magnitude of *M* and sign of $\gamma _{0}$. We have produced the phase diagram between the BMF interaction amplitude and the oscillator frequency for a better insight of droplet-soliton transition. Utilizing the elliptic solution of eGPE and oscillator frequency, we demonstrates a novel mechanism for fragmentation, merging and transport of QDs. The stability of obtained wavefunction solutions are confirmed using VK criterion.

As an extension of the present work, it may be interesting to verify the collision of small/large droplets, its impact on fragmentation/merger and study its dynamics in 2D/3D dimensions in presence of harmonic trap.

## Methods

We consider the Bose-Bose mixture BEC with equal masses and equal number of atoms in the components under the influence of BMF (LHY corrections for quantum fluctuations) in presence of temporally varying external harmonic confinement. By taking the spinor components of binary mixture as mutually symmetric, the Hamiltonian is taken as

21 $$\begin{aligned} i\frac{ \partial \psi }{\partial t} =- \frac{1}{2} \frac{ \partial ^2 \psi }{\partial x^2} - g_1(t) |\psi |\psi + g_2(t)|\psi |^2 \psi + \frac{1}{2}M(t)x^2 \psi . \end{aligned}$$

i.e. in the form of time-dependent dimensionless 1D eGPE. Using similarity transformation technique, we assume an ansatz solution and connect the 1D eGPE with a solvable PDE. This results into a number of constraints over phase, MF and BMF nonlinearities as PDEs. Next, we solve these equations and obtain the general form of wavefunction solution.

## Data Availability

All data generated or analysed during this study are included in this published article. It can be reproduced by utilizing the form of wavefunction and considered trap form.
